# Redescription of the Advertisement Call of Five Species of *Thoropa* (Anura, Cycloramphidae), Including Recordings of Rare and Endangered Species

**DOI:** 10.1371/journal.pone.0162617

**Published:** 2016-09-12

**Authors:** Carlos H. L. Nunes-de-Almeida, Clodoaldo L. Assis, Renato N. Feio, Luís Felipe Toledo

**Affiliations:** 1 Laboratório de História Natural de Anfíbios Brasileiros (LaHNAB), Departamento de Biologia Animal, Instituto de Biologia, Unicamp, Campinas, São Paulo, Brazil; 2 Universidade Federal de Viçosa, Departamento de Biologia Animal, Museu de Zoologia João Moojen, Viçosa, Minas Gerais, Brazil; University of Pavia, ITALY

## Abstract

Frogs of the genus *Thoropa* comprise six endemic Brazilian species on the Eastern side of the country. Little is known about their natural history, especially about their acoustic communication. Therefore, aiming to provide an overview of their vocalizations, we analyzed and redescribed male advertisement calls of three living and two possibly extinct species. The smaller species, *T*. *petropolitana* and *T*. *lutzi*, produce simple calls (one single note) with a higher frequency range than the remaining larger ones. On the other hand, the larger species present complex calls, with more than one note: *T*. *megatympanum* calls have three notes, *T*. *taophora* calls have four notes, and *T*. *miliaris* calls varies from three to six notes. Population snout-vent length negatively correlated with peak of dominant frequency as expected. However, highlighted differences between two populations of *T*. *lutzi*, which could indicate need of further taxonomic evaluation of those lineages. Peculiar morphology, such as the absence of vocal sacs and slits, may have contributed to their call variation and highly banded frequency structure. If the observed population differences reflect species-level differences, *T*. *lutzi* may be classified as a critically endangered species, as *T*. *petropolitana*. Furthermore, we provided a suggestion to an unusual behavior in frogs: calling with the mouth open in the smaller species of the genus.

## Introduction

General interest in anuran acoustic communication is widespread as vocalizations are important for taxonomy (e.g. [1, 2]), natural history (e.g. [3, 4]), and evolutionary centered studies (e.g. [5, 6]). In spite of that, call descriptions are lacking for most anuran species worldwide (L. F. Toledo, unpublished data), hindering advances in their classification. Anuran calls are produced almost exclusively by males (see exceptions in [7]), being mainly associated with mate attraction [8]. There is strong selection acting on advertisement calls being subject to both sexual and natural selection [9, 10, 11, 12]. Therefore, recent studies [13, 14] showed that there is little variation among populations of the same species.

**Fig 1:**
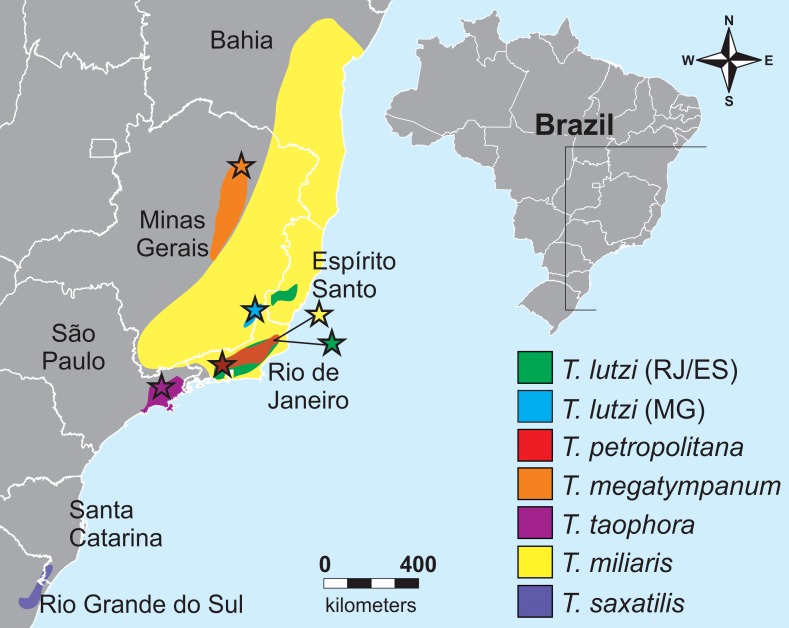
Geographic distribution of the species of the genus *Thoropa* in Eastern Brazil. Modified from IUCN [29] distribution maps. Stars indicate localities where advertisement call recordings were made.

Frogs of the genus *Thoropa*, currently allocated in the family Cycloramphidae, includes six species: *T*. *miliaris* ([15]), *T*. *petropolitana* ([16]), *T*. *taophora* ([17]), *T*. *lutzi* [18], *T*. *megatympanum* [19], and *T*. *saxatilis* [20] [19, 20, 21, 22]. They are endemic to Eastern Brazil, occurring from the state of Bahia to the state of Rio Grande do Sul, occupying rocky fields of Atlantic Forest (mainly) and Cerrado [21, 23] (Fig 1). These species are small to medium sized (28–102 mm) and occur from sea level to about 1500 m of elevation [20, 21, 22]. They usually call during the nights of summer [20, 23] and their dorsal coloration was selected to resemble rocks and the surrounding background where they live [24]. At least *T*. *taophora* is polygamous, with recent observations showing two females sharing breeding sites [25]. They can stand salty water on rocky shores where they prey on marine invertebrates [26, 27]. As an adaptation to the marine environment, these animals have higher osmotic plasma, musculature, and urine concentration [27]. *Thoropa* spp. lay eggs on rocky walls with a thin layer of flowing water. After hatching, tadpoles are semi-terrestrial [23, 28] and could be cannibalistic [25]. Therefore, the genus is unique in several aspects from the ecological point of view.

Heyer [30] mentioned that *T*. *petropolitana* was common on rocky cliffs, at Serra dos Órgãos, Teresópolis, Rio de Janeiro. However, it has vanished from this location. Caramaschi *et al*. [31] referred to the decline and disappearance of *T*. *petropolitana*, and it is currently endangered (EN) in the red list of the state of Rio de Janeiro [31] and in the current national red list [32]. *Thoropa lutzi* has also disappeared from Serra dos Órgãos since 1979 and is currently categorized as data deficient (DD) in the state of Rio de Janeiro and national lists [31, 32]. In the state of Espírito Santo *T*. *lutzi* is endangered (EN). Besides these later two syntopic species, *T*. *saxatilis* from southern Brazil is currently classified as endangered (EN) by the state and vulnerable (VU) by the national red list [32, 33]. The other three known species are not under extinction risk. Therefore, the genus is also unique in the sense that half of its known diversity is endangered (Table 1).

**Table 1:** Conservation status of the species of the genus *Thoropa* in accordance to distinct sources. The IUCN Red List of Threatened Species [29], Brazilian official red list [32], and red lists of the state of Espírito Santo (ES) [34], Minas Gerais (MG) [35], Rio de Janeiro (RJ) [31], Rio Grande do Sul (RS) [33], and São Paulo (SP) [36]. Categories are least concern (LC); near threatened (NT); vulnerable (VU); endangered (EN); critically endangered (CR); data deficient (DD); and not evaluated (NE).

			States				
Species	IUCN	Brazil	ES	RJ	MG	SP	RS
***T*. *lutzi***	EN	DD	EN	CR	-	-	-
***T*. *megatympanum***	LC	LC	-	-	LC	-	-
***T*. *miliaris***	LC	LC	LC	LC	LC	NE	-
***T*. *petropolitana***	VU	EN	CR	EN	-	-	-
***T*. *saxatilis***	NT	VU	-	-	-	-	EN
***T*. *taophora***	NE	LC	-	-	-	LC	-

In spite of that, detailed information about their vocalizations is still lacking. There is nothing besides brief and old descriptions of the advertisement calls of *T*. *miliaris*, *T*. *petropolitana*, and *T*. *lutzi* [37]. Heyer *et al*. [38] characterized its advertisement of *T*. *taophora*, at that time identified as *T*. *miliaris* [22], as short, low-pitched, noisy, and composed of several complexly pulsed notes. Caramaschi and Sazima [20] reported that the advertisement call of *T*. *megatympanum* is similar to that of *T*. *miliaris*. Finally, Feio [25] provided information on the advertisement call of this species, but based on recordings made in Serra do Cipó, state of Minas Gerais. The call of *T*. *saxatilis* remains unknown, with no recordings or references available about its vocalization.

Due to lack of detailed information about the vocalizations of the congeneric species, and the necessity of standardization that allows comparisons of other acoustic variables, we re-described the advertisement calls of five out of six species of the genus in detail, including new populations of endangered and apparently extinct species. These results will complement the existing information in the literature, and facilitate future advances of their behavioral ecology and phylogenetic relationships.

## Materials and Methods

We examined specimens from Brazilian museums to acquire information about their snout-vent length (SVL): Museu de Zoologia “Prof. Adão José Cardoso”, Unicamp, Campinas, São Paulo (ZUEC); Célio F. B. Haddad Amphibian Collection, Departamento de Zoologia, Unesp, Rio Claro, São Paulo (CFBH); Museu Nacional, Rio de Janeiro (MNRJ); and Museu de Zoologia João Moojen de Oliveira, Universidade Federal de Viçosa, Minas Gerais (MZUFV) (S1 File).

Advertisement calls were obtained from Fonoteca Neotropical Jacques Vielliard, Unicamp, Campinas, São Paulo (FNJV), and the Smithsonian Institution website for an additional call of *T*. *taophora* from Boraceia [38]. To the present date and our knowledge, there are no other recordings of these species in other sound archives. All individuals recorded were adult males (females are not reported to call in this genus), being one male per species, except for the population of *T*. *lutzii* from the state of Minas Gerais, for which we had access to recordings of two males (5 and 25 calls from each; FNJV 32325 and 32324, respectively). We accessed the same recordings used by Bokermann [37] for descriptions of advertisement call of *T*. *miliaris*, *T*. *petropolitana*, and *T*. *lutzi* in the state of Rio de Janeiro and the same calls analyzed by Heyer *et al*. [38] for *T*. *taophora*. Additionally, we used recent recordings for *T*. *megatympanum* and *T*. *lutzi* of the state of Minas Gerais. All these recordings were made using different equipment (see Table 2 for complete information on the recordings) and were digitized with a sample rate of 96 kHz, at 16 bits of resolution, mono and wave format file.

**Table 2:** Equipment and sound analyses settings used for the recordings of six populations of five *Thoropa* species. n/a = data not available.

Data		*T*. *megatympanum*	*T*. *miliaris*	*T*. *taophora*	*T*. *petropolitana*	*T*. *lutzi*	
						Minas Gerais	Rio de Janeiro
**Recording information**	**Recorder**	Marantz PMD222	Uher 4000 Report IC	n/a	Uher 4000 Report IC	Tascam DR40	Uher 4000 Report IC
	**Microphone**	Audio-Technica AT835b	Electronic Parabolic Microphone P200	n/a	Electronic Parabolic Microphone P200	Sennheiser ME66/K6	Electronic Parabolic Microphone P200
	**Microphone frequency range**	0.04–20 kHz	0.35–15 kHz	n/a	0.35–15 kHz	0.04–20 kHz	0.35–15 kHz
	**Recording speed**	4.76 cm/s	9.5 cm/s	n/a	9.5 cm/s	–	9.5 cm/s
	**Date of record**	01 December 2005	25 January 1964	December 1976	23 May 1963	01 January 2016	25 January 1964
	**Recordist**	L. F. Toledo	W. C. A. Bokermann	W. R. Heyer	W. C. A. Bokermann	C. L. Assis	W. C. A. Bokermann
	**Catalog number **	FNJV 31421	FNJV 31770	USNM 209326	FNJV 31759	FNJV 32324–25	FNJV 31772
**Analyses information**	**Brightness**	65	42	76	43	45	45
	**Contrast**	74	62	78	58	65	65
	**FFT**	512	256	256	256	256	512
	**Window type**	Hann	Hann	Hann	Hann	Hann	Hann
	**Window size**	512	256	256	256	256	512
	**Bandwidth**	270 Hz	539 Hz	539 Hz	539 Hz	539 Hz	270 Hz
	**Overlap**	50%	50%	50%	50%	50%	50%
	**Hop size**	256	128	128	128	128	256
	**DFT size**	512	256	256	256	256	512
	**Grid spacing**	188 Hz	375 Hz	375 Hz	375 Hz	375 Hz	188 Hz

We analyzed the recordings in Raven Pro 64 1.4 (Cornell Lab of Ornithology) using different settings, as these calls were recorded with different recorders and at probably different distances (normally around 50 cm to 1 m from the calling male). These settings are summarized in Table 2. We selected the following bioacoustical characters to describe the calls: 1) duration (obtained by the function “delta time” in Raven); 2) number of pulses per note; 3) number of notes per call (counted visually); 4) call duration; 5) pulse duration; 6) interval between calls (all estimated via delta time function); 7) a proxy of the lower frequency (using “Frequency 5%” function); 8) a proxy of the maximum frequency (using “Frequency 95%” function)–these last both measures include maximum and minimum frequencies, ignoring 5% below and above of total energy in the selected call; 9) peak of dominant frequency (using the function “Max Frequency”–the frequency in which the power is maximum within the call). Besides these, we evaluated presence of harmonics and modulation of frequencies (analyzed visually). Temporal information was obtained from the oscillogram and spectral information from the spectrogram. We did a linear regression analysis to verify the possible influence of SVL in the peak of dominant frequency of the advertisement calls of all studied populations.

Based on bioacoustic terminology reviewed by Toledo *et al*. [6], we applied: 1) call–as the anuran vocalization that include one or more notes; 2) note–pulsed or tonal vocalization units that compose a call; 3) pulse–subunits of a note, the smallest indivisible part of a note; and 4) harmonics–The signal component of the frequency which is an integer multiple of the fundamental frequency. Additionally, we hereby define 5) frequency modulation–as the modulation of frequency bands, which, when present, can be ascending (from lower to higher frequency) or descending (on the opposite direction); and 6) pseudopulse–distinguishable set of pulses, but which are not characterized as one note, because they are not temporally discrete.

## Results

**Fig 2:**
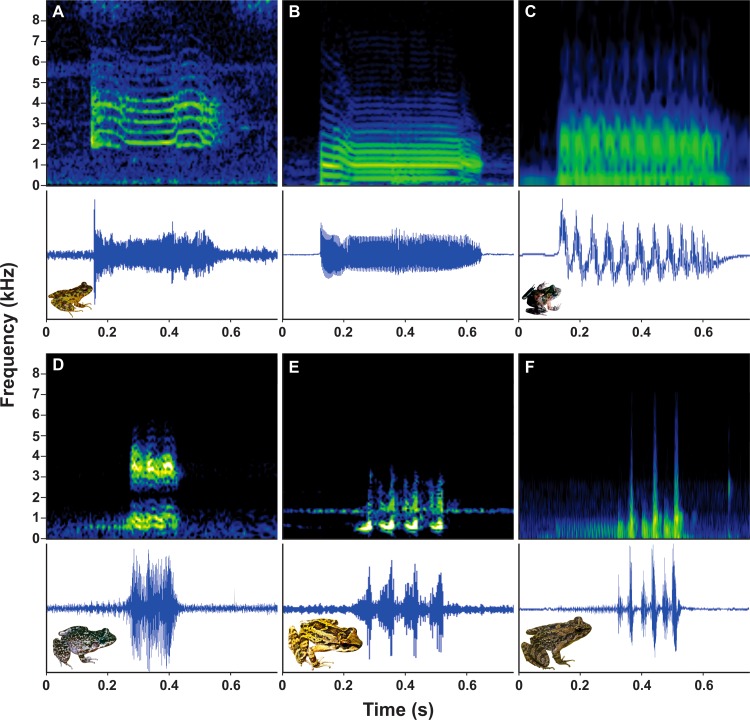
Spectrogram (above) and oscillogram (below) of the advertisement calls of five species of *Thoropa*. (A) Advertisement calls of *T*. *lutzi* from the state of Minas Gerais; (B) *T*. *lutzi* from the type locality in the state of Rio de Janeiro; (C) *T*. *petropolitana* from state of Rio de Janeiro; (D) *T*. *megatympanum* from the state of Minas Gerais; (E) *T*. *taophora* from the state of São Paulo; and (F) *T*. *miliaris* from the type locality in the state of Rio de Janeiro.

Advertisement calls of *T*. *lutzi* (RJ and MG) and *T*. *petropolitana* (Fig 2A–2C) have only one note (= simple calls). Calls of *T*. *lutzi* (Fig 2A and 2B) present harmonics and the other species (Fig 2C–2F) have calls with pulsatile nature. The call of the *T*. *lutzi* (Fig 2A) can be divided into three portions, the initial with ascend and descend frequency modulations, the middle without modulation, and the final portion again with ascending and descending modulations. The call of *T*. *lutzi* (Fig 2B) presents an initial portion with descending modulation, the middle without modulation, and the final with descending modulation. The calls of the remaining larger species are composed (= more than one note/call): *T*. *megatympanum* has three identical notes; *T*. *taophora* has three to four notes; and *T*. *miliaris* ranging four to six notes (Fig 2D–2F).

**Fig 3:**
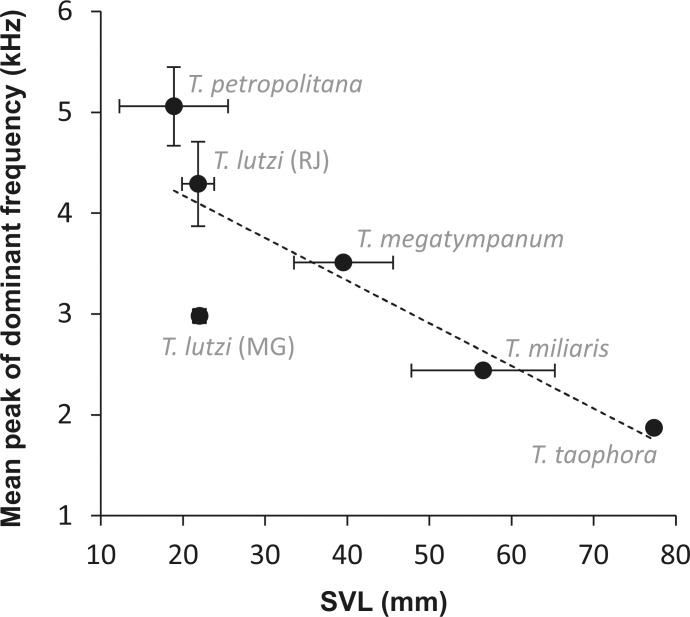
Linear regression between *Thoropa* snout-vent length (SVL) and peak of dominant frequency. Horizontal bars represent the SVL range and verticals lines represent the dominant frequency range.

The linear regression analysis negatively related (F_(4,1)_ = 9.65; r^2^ = 0.71; *P* = 0.03) the body mass with the peak of dominant frequency (Fig 3).

### Thoropa lutzi

The advertisement call of two individuals from state of Minas Gerais were single (with one note) and short, with the mean duration of 279 ± 22 ms (232–316), and mean interval between calls of 85 ± 56.2 s (40.5–241.4). The call had a harmonic structure with frequency modulation, presenting up to 10 harmonics. The peak of dominant frequency coincided with the first harmonic in 64% the calls, with a mean frequency of 2.17 ± 0.08 kHz (2.06–2.23), fourth harmonic (3% of the calls) with a frequency of 4.47 kHz (n = 1), and varying between the first and second harmonics (33% of the calls) with mean frequency of 2.49 ± 0.11 kHz (2.41–2.75). The mean minimum frequency in the advertisement call was 2.00 ± 0.08 kHz (1.89–2.06) and the maximum frequency was 4.77 ± 0.57 kHz (4.30–6.54) (Table 3; Fig 2A).

**Table 3:** Advertisement call parameters and size of species of genus *Thoropa*. Values presented as mean ± standard deviation (range; sample size). When only one value is presented the number of calls is equal to 1. Two populations of *T*. *lutzi* are present, one from the state of Minas Gerais and another from the type locality in the state of Rio de Janeiro.

Parameter / Species	*T*. *megatympanum*	*T*. *miliaris*	*T*. *taophora*	*T*. *petropolitana*	*T*. *lutzi*	
					Minas Gerais	Rio de Janeiro
**SVL (mm)**	39.74 ± 6.62 (28.73–51.28; 20)	56.54 ± 6.04 (47.00–68.44; 6)	77.35 ± 8.73 (58.93–92.11; 27)	18.88 ± 1.98 (15.15–21.92; 20)	22 ± 0.81 (20.7–23.5; 16)	21.83 ± 0.78 (20.83–23.40; 14)
**Call duration (ms)**	168 ± 0.17 (145–184; 5)	339 ± 0.42 (261–378; 5)	257 ± 0.34 (223–291; 2)	36 ± 1 (34–37; 6)	279 ± 22 (232–316; 30)	196 ± 5 (185–203; 7)
**Pulse duration (ms)**	3 ± 0.4 (2–4; 10)	1 (1–1; 10)	2 (2–2; 10)	3 ± 0.4 (2–3; 10)	–	–
**Peak of dominant frequency (kHz)**	2.411 ± 1.03 (1.033–3.445; 5)	2.137 ± 0.09 (2.062–2.250; 5)	0.750 (0.750–0.750; 2)	3.812 ± 0.40 (3.000–4.125; 6)	2.36 ± 0.43 (2.06–4.47; 30)	1.723 (1.723–1.723; 7)
**Minimum frequency (kHz)**	0.792 ± 0.15 (0.517–1.033; 5)	0.187 (0.187–0.187; 5)	0.375 (0.375–0.375; 2)	2.562 ± 0.26 (2.250–3.000; 6)	2.00 ± 0.08 (1.89–2.06; 30)	0.812 ± 0.15 (0.517–1.033; 7)
**Maximum frequency (kHz)**	3.514 ± 0.39 (2.756–3.789; 5)	2.437 (2.437–2.437; 5)	1.875 (1.875–1.875; 2)	5.062 ± 0.42 (4.500–5.625; 6)	4.77 ± 0.57 (4.30–6.54; 30)	2.977 ± 0.07 (2.928–3.100; 7)
**Notes per call**	3 (3–3; 5)	5.4 (4–6; 5)	3.5 (3–4; 2)	1 (1–1; 6)	1 (1–1; 30)	1 (1–1; 7)
**Pulses per note in the first note (only measured for *T*. *miliaris*)**	–	170.6 ± 44.46 (87–212; 5)	–	–	–	–
**Pulses per note**	132.1 ± 29.37 (60–186; 10)	46.2 ± 12.75 (33–76; 10)	52.57 ± 6.34 (42–62; 7)	132.33 ± 8.94 (118–145; 6)	–	–
**Pseudopulses per call (only measured for *T*. *petropolitana*)**	–	–	–	11.17 ± 0.37 (11–12; 6)	–	–
**Interval between calls (s)**	10.96 ± 0.34 (10.50–11.43; 4)	10.20 ± 15.41 (0.23–36.82; 4)	6.02	23.30 ± 5.88 (14.64–31.54; 5)	85.0 ± 56.2 (40.5–241.4; 28)	23.76 ± 11.78 (9.42–46.02; 6)
**Air temperature (°C)**	20	20	19	14	23.5	20
**Coordinates**	16º37'S, 42º50'W	21º41'S, 41º33'W	23º38'S, 45º52'W	22º30'S, 43º10'W	20º58'S, 42º10'W	21º41'S, 41º33'W
**Municipality, state**	Grão Mogol, MG	Sumaré, RJ	São Sebastião, SP	Petrópolis, RJ (Type locality)	Antônio Prado de Minas, MG	Sumaré, RJ (Type locality)

The advertisement call of the individual from Morro do Sumaré, municipality of Rio de Janeiro, was single and had a mean duration of 196 ± 5 ms (185–203), mean interval between calls of 23.7 ± 11.7 s (9.4–46). The call presented up to 23 harmonics, with frequency modulation. The peak of the dominant frequency coincided with the initial portion of second harmonic and the middle portion of the third harmonic in 1.72 kHz. The mean minimum frequency was 0.81 ± 0.15 kHz (0.51–1.03), and mean maximum frequency was 2.98 ± 0.07 kHz (2.93–3.10) (Table 3; Fig 2B).

### Thoropa petropolitana

Its advertisement call had a mean duration of 36 ± 1 ms (34–37), mean interval between calls ranging from 14.64 to 31.54 s and repeated at irregular intervals. Each call consists of a single note with a mean of 132.33 ± 8.94 pulses per note (118–145). Pulses had a mean duration of 3 ± 0.4 ms (2–3), and were grouped in pseudopulses with a mean of 11.17 ± 0.37 pulses (11–12). The mean peak of dominant frequency was 3.81 ± 0.4 kHz (3.00–4.12), mean minimum frequency was 2.56 ± 0.26 kHz (2.25–3.00), and mean maximum frequency was 5.06 ± 0.42 kHz (4.50–5.62). This call had three frequency bands without frequency modulation (Table 3; Fig 2C).

### Thoropa megatympanum

The advertisement call of *T*. *megatympanum* resembles the call of *T*. *taophora* and *T*. *miliaris*, but was shorter and repeated at shorter intervals. The call had a mean duration of 168 ± 0.17 ms (145–184), mean interval between calls of about 10.96 ± 0.34 s (10.50–11.43), and each call consisted of three notes with a mean of 132.1 ± 29.37 pulses per note (60–186). The mean pulse duration was 3 ± 0.4 ms (2–4), mean peak of dominant frequency was 2.41 ± 1.03 kHz (1.03–3.44), mean minimum frequency was 0.79 ± 0.15 kHz (0.51–1.03), and mean maximum frequency was 3.51 ± 0.39 kHz (2.76–3.79). This call had two frequency bands without frequency modulation (Table 3; Fig 2D).

### Thoropa taophora

The advertisement call was composed of three to four notes with a mean of 52.57 ± 6.34 pulses per note (42–62), with mean pulse duration of 2 ms. The mean duration of call was 257 ± 0.34 ms (223–291) and the mean interval between the calls was 6.02 s. The peak of dominant frequency was 0.75 kHz, mean minimum frequency was 0.37 kHz, and the mean maximum frequency was 1.87 kHz. This call had two frequency bands without frequency modulation (Table 3; Fig 2E).

### Thoropa miliaris

The advertisement call was composed of four to six notes. The first note with a mean of 170.6 ± 44.46 (87–212) pulses per note, and the other notes with a mean of 46.2 ± 12.75 (33–76) pulses per note, with mean pulse duration of 1 ms. The mean call duration was 339 ± 0.42 ms (261–378) and the mean interval between the calls was 10.2 ± 15.41 s (0.23–36.82). The mean peak of dominant frequency was 2.14 ± 0.09 kHz (2.06–2.25), mean minimum frequency is 0.19 kHz, and mean maximum frequency was 2.44 kHz. This call had only one frequency band without frequency modulation (Table 3; Fig 2F).

## Discussion

Even though the number of advertisement calls per species is low, we were able to detect differences, both in temporal, spectral, and structural parameters between the smaller (*T*. *petropolitana* and *T*. *lutzi*; smaller than 24 mm in SVL) and the remaining larger *Thoropa* species (larger than 28 mm in SLV). These differences could be biased due to our limited sample, or could indeed reflect possible natural relationships between these two phenetic groups. Hereby we highlight the difference between simple and complex calls, and the presence and absence of harmonic structure as binary characters, which may improve subsequent and yet unavailable phylogenetic hypotheses for the genus [39]. Phylogenetic reconstructions using anuran advertisement call characters [39] can corroborate the results of morphological and molecular analyzes [40, 41]. In agreement, some acoustic characters may contain significant phylogenetic signal [41, 42], indicating possible applicability of such characters.

Species of the genus *Thoropa* does not have sacs or slits [43]. The absence of these structures probably influence the sound produced by these anurans [44]. The advertisement calls of the smaller *Thoropa* species are similar in structure (i.e., presence of several harmonic bands, reaching frequencies over 5 kHz) to anuran defensive screams [45] and to the calls of anuran species that vocalize with the mouth open (e.g. [46, 47, 48, 49]). We are not aware about the mechanistics of the production of these advertisement calls. However, its harmonic structure may be related to opening the mouth while calling, suggestively, in order to compensate for the absence of vocal slits and sacs. We further based our suggestions in that males of *T*. *taophora* have been reported to call with their mouth open during male-male aggressive interactions [43]. Confirmation of this hypothesis is important, as it would help us to elucidate the relationship between the larger and smaller species of the genus.

Differences between the calls of the two populations of *Thoropa lutzi* highlight the need of a closer taxonomic evaluation. In spite of our limited sample, ranges of the peak of dominant frequencies are clearly different between the population of Rio de Janeiro and Minas Gerais, in spite of the overlapping SVL. Peak of dominant frequencies is generally conservative in anurans [41, 42] and is frequently used as a good taxonomic character [50, 51], even when species are morphologically cryptic [52, 53]. Therefore, these populations of *T*. *lutzi* may represent different lineages, requiring further investigation for adequate classification. If these two populations are indeed distinct species, *T*. *lutzi* may be restricted to the living population of Espírito Santo; which also could be distinct from the vanished Rio de Janeiro type population. If these differences really indicate specific differences (not only populational ones), the threatened status of *T*. *lutzii* will change to one of the threatened categories, probably CR as *T*. *petropolitana*. Therefore, our study showed to be relevant to various aspects, contributing to future taxonomy, conservation assessment, and behavioral ecology (given that it indicates possible unusual calling behavior in an Atlantic forest anuran).

## Supporting Information

S1 File List of the adult individuals analyzed for snout-vent length. (PDF)
