# Supplementary material for: Redescription of the Advertisement Call of Five Species of Thoropa (Anura, Cycloramphidae), Including Recordings of Rare and Endangered Species
Source: PLoS One. 2016 Sep 12;11(9):e0162617. doi: 10.1371/journal.pone.0162617 (PMC5019406; doi:10.1371/journal.pone.0162617)
Supplement: S1 File — (PDF) [file pone.0162617.s001.pdf]

S1 File. List of the adult individuals analyzed for snout-vent length.

Thoropa taophora (25 individuals): CFBH 10417 Ilha Bela - São Paulo; ZUEC 14, 629, 899, 900, 1105, 1111, 1274, 1336, 1340, 1484, 1512, 1519, 1528–29, 1532, 1537, 1738, 1745, 1858, 2020, 2141, 2625, 2627, 9007 Ubatuba - São Paulo.

Thoropa miliaris (20 individuals): CFBH 25110–11, 25117 Domingos Martins - Espírito Santo; 26379 Linhares - Espírito Santo; MNRJ 5831 Teresópolis - Rio de Janeiro; 9347, 9350 Ilha Mangaratiba - Rio de Janeiro; 23530, 23587 Sinonésia - Minas Gerais; 22866, 26499–500 Rio de Janeiro - Rio de Janeiro; 27500–01 Braga - Minas Gerais; 46861 Itamaraju - Bahia; 53500 Cachoeira de Macacu - Rio de Janeiro; 76758, 76760, 84656 Saquarema - Rio de Janeiro; 84073 Santa Tereza - Espírito Santo.

Thoropa megatympanum (20 individuals): CFBH 789, 791 Santana do Riacho - Minas Gerais; 10194, 10196 Grão Mogol - Minas Gerais; MNRJ 26160, 26162–63 Jaboticatubas - Minas Gerais; 22906–09, 22913 Botumirim - Minas Gerais; MZUFV 4115 Jaboticatubas - Minas Gerais; ZUEC 2102, 2202, 2319 Santana do Riacho - Minas Gerais; 2841–42, 3957, 15938 Jaboticatubas - Minas Gerias.

Thoropa petropolitana (20 individuals): MNRJ 23060, 25950–51, 25954–56 Teresópolis - Rio de Janeiro; 23344, 23351, 23355–360 Petrópolis - Rio de Janeiro; 24148–49, 24151–53, 24158 Tinguá - Rio de Janeiro.

Thoropa lutzi (14 individuals): MNRJ 1373 Santa Tereza - Espírito Santo; MZUFV 13820–29, 13837–39 Cataguases e Prado de Minas - Minas Gerias.
